# Whole-genome sequencing and characterization of human fecal isolate *Lacticaseibacillus casei* LC130 from NORDBIOTIC collection

**DOI:** 10.1128/mra.00507-24

**Published:** 2024-07-02

**Authors:** Agnieszka K. Szczepankowska, Bożena Cukrowska, Tamara Aleksandrzak-Piekarczyk

**Affiliations:** 1 Institute of Biochemistry and Biophysics, Polish Academy of Sciences, Warsaw, Poland; 2 Department of Pathomorphology, The Children Memorial Health Institute, Warsaw, Poland; Portland State University, Portland, Oregon, USA

**Keywords:** probiotics, prohealth effects, bacteriocins, gluten-degrading peptidases, polyamines, lactose metabolism, lactic acid bacteria, fecal human isolate

## Abstract

We report the complete genome sequence of *Lacticaseibacillus casei* LC130, isolated from a healthy human fecal sample and part of the NORDBIOTIC collection. The 2.969 Mb genome of LC130 includes genes potentially involved in lactose metabolism and the production of bacteriocins, peptidases, and polyamines, suggesting potential health benefits.

## ANNOUNCEMENT

*Lacticaseibacillus casei* LC130, a lactic acid bacterium, was isolated from a healthy human fecal sample and included in the NORDBIOTIC collection, deposited in the Leibniz Institute DSMZ-German Collection of Microorganisms and Cell Cultures GmbH (DSM33796). LC130 alleviates symptoms in patients with irritable bowel syndrome (IBS) (1) and acute respiratory tract infections (2), and reduces immunoreactivity of gluten-derived peptides (3).

The strain, provided by Nordic Biotic Ltd. (Warsaw, Poland) as part of the NORDBIOTIC collection, was cultured at 37°C in De Man, Rogosa and Sharpe medium (Oxoid) under aerobic conditions. DNA was extracted using the cetyltrimethylammonium bromide/lysozyme method (4). Bacterial cell walls were digested with lysozyme (20 mg/mL; Sigma-Aldrich) and mutanolysin (5 U/mL; A&A Biotechnology) (15 min, 37°C). DNA concentration was measured using a Qubit fluorimeter (Thermo Fisher Scientific) and used for both Illumina and Nanopore sequencing.

Illumina libraries were constructed with the NEB Ultra II FS kit (New England Biolabs) and sequenced in paired-end mode (v.3, 600 cycle chemistry kit) on a MiSeq equipment (Illumina). Quality was checked using FASTQC (v.0.12.0) (5), and short reads were filtered using fastp (v.0.23.2) (6).

For long-read sequencing, DNA was sheared with a 26G needle, and fragments shorter than 10 kb were excluded using the Short Read Eliminator kit (Circulomics). A 1D library was constructed with the SQK-LSK109 native barcoding expansion kit (EXP-NBD103) and sequenced using an R9.4.1 flowcell on a GridION sequencer (Oxford Nanopore Technologies). Raw Oxford Nanopore Technology (ONT) reads were base-called using Guppy (v.6.1.3) (https://github.com/nanoporetech/pyguppyclien) in super accuracy mode. Short (<1 kb) and low quality (average quality score < 12) reads were removed by NanoFilt (v.2.8.0) (7) and adapters were trimmed with Porechop (v.0.2.4) (https://github.com/rrwick/Porechop). Data quality check was done with NanoPlot (v.1.41.6) (7). ONT long-reads were assembled with Trycycler (v.0.5.3) (8) and Flye (v.2.9) (9), Unicycler (v.0.4.8) (10), Raven (v.1.8.1) (11) and Miniasm (v.0.3-r179) (12), and polished using Racon (v.1.5.0) (13) and Medaka (v.1.7.2) (14). Circularization and contig rotation were attained by Trycycler (v.0.5.3) and verified by Bandage (v.0.8.1) (15) and Tablet (v.1.17.08.17) (16). Default parameters were used for all software. ONT and Illumina assemblies were combined and polished using Polypolish (v.0.5.0) (17) and POLCA (v.4.0.5) (18). The final coverage for the chromosome was 224x and for plasmid 1373x. Illumina and ONT sequencing statistics are presented in Table 1.

**TABLE 1:** Short- and long-read data statistics for *L. casei* LC130 genome sequencing

Contig	Illumina sequencing			ONT sequencing			
	Number of reads	sequence data (nt)	coverage	Number of reads	sequence data (nt)	N50	coverage
chromosome	596,564	270,245,401	88x	33,879	434,236,279	12,410	136x
plasmid pLC130_p1			267x				1106x

The genome revealed a circular chromosome (2,940,740 bp, 47.9% GC) and a circular plasmid (28,032 bp, 44.08% guanine-cytosine), with 2,898 predicted coding sequences, 59 tRNAs, 15 rRNAs, and 3 ncRNAs. Annotation using the NCBI Prokaryotic Genome Annotation Pipeline (v.6.6) (19) identified genes encoding enzyme IIA and enzyme IICB—components of the lactose-specific phosphotransferase system, a phospho-β-galactosidase, and the complete tagatose pathway. Strong acidifying properties may enhance calcium/iron solubility and absorption by intestinal cells, increasing bone calcium content and alleviating anemia (20 – 22). Four potential class IId bacteriocins and a gene encoding ornithine decarboxylase, converting ornithine to putrescine (a beneficial polyamine), were found, which may tighten intestinal cell junctions (23 – 26), contributing to IBS symptom relief (1). Genes encoding peptidases that could degrade immunogenic gluten-derived peptides were also present (3).

## Data Availability

Complete sequencing data has been deposited in GenBank under BioProject: PRJNA1059784; BioSample: SAMN39212590. Whole genomic data is available under accession numbers CP143264 (chromosome) and CP143265 (pLC130_p1). lllumina SRA reads are available under accession number SRR27494201. Oxford Nanopore SRA reads are available under accession number SRR27494202.
